# Supplementary material for: Role of Splicing Regulatory Elements and In Silico Tools Usage in the Identification of Deep Intronic Splicing Variants in Hereditary Breast/Ovarian Cancer Genes
Source: Cancers (Basel). 2021 Jul 3;13(13):3341. doi: 10.3390/cancers13133341 (PMC8268271; doi:10.3390/cancers13133341)
Supplement: Supplementary file 1 [file cancers-13-03341-s001.zip › Supplementary figures.pdf]

cancers

MDPI

Supplementary Materials: Role of Splicing Regulatory Elements and In Silico Tools Usage in the Identification of Deep Intronic Splicing Variants in Hereditary Breast/Ovarian Cancer Genes

Alejandro Moles-Fernández, Joanna Domènech-Vivó, Anna Tenés, Judith Balmaña, Orland Diez and Sara Gutiérrez-Enríquez

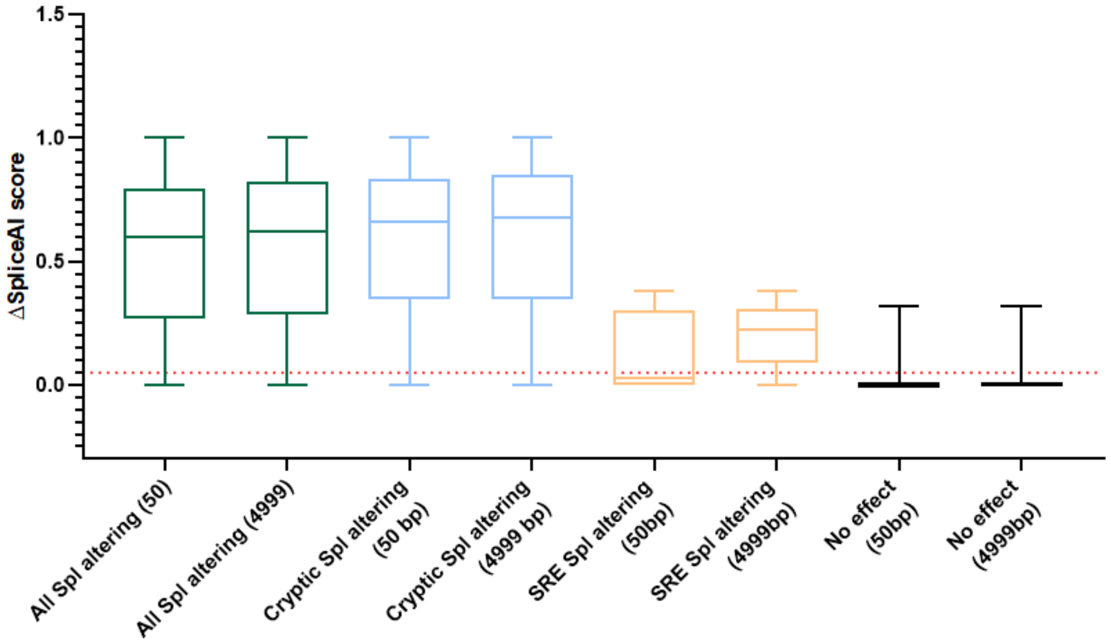

Figure S1. ΔSpliceAI scores of spliceogenic and not spliceogenic variants collected in the literature database, taking predictions of cryptic sites in a window of 50bp or 4999bp. In the analysis with the 16 splicing altering variants by affecting SREs, the ΔSpliceAI evaluation of the 4999 bp located on each side of the variant reached higher predictive power in comparation with a 50 bp window analysis. Red line indicates a 0.05 score cut-off, and a mean ± standard deviation are represented in each boxplot graph.

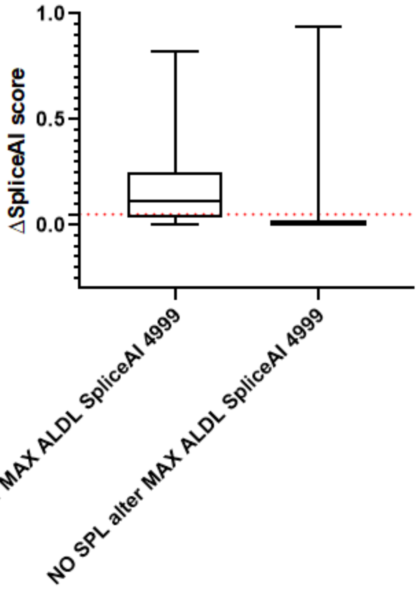

Figure S2. Comparison of ΔSpliceAI scores between spliceogenic and not spliceogenic exonic variants collected from Tubeuf et al., 2020. ΔSpliceAI values of the 4999 bp located on each side of the variant in spliceogenic ones reach higher scores comparing with not spliceogenic variants. Red line indicates a 0.06 score cut-off , and a mean ± standard deviation are represented in each boxplot graph. MAX ALDL, maximun value of Acceptor Loss and Donor Loss of SpliceAI.

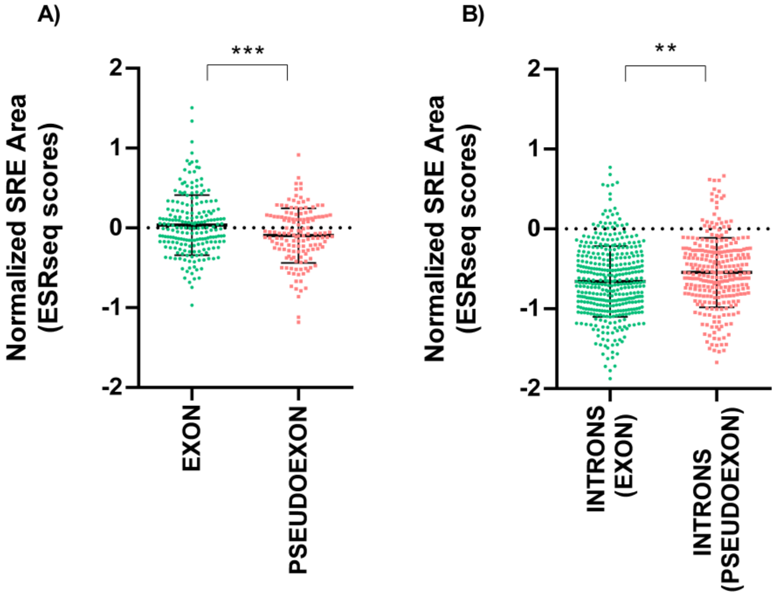

Figure S3. Comparison of SREs abundance in different genomic regions, using the Normalized SRE Area calculated using ESRseq scores. (**A**) Differences in the normalized area ESRseq scores of regulatory elements between canonical exons and pseudoexons. A greater abundance of positive ESRseq values was observed in canonical exons (*t*-test, ****p*-value< 0.001). (**B**) Differences in the normalized area ESRseq scores of regulatory elements between introns adjacent to canonical exons and pseudoexons. A mean was calculated with the two values of normalised area SRE of the acceptor or donor flanking intronic regions of each exon/pseudoexon. A greater abundance of negative ESRseq values was observed in introns adjacent to canonical exons (*t*-test, ***p*-value ≤ 0.01). Mean ± standard deviation is represented in each graph.
